# Natural soundscapes, urban design and psychological well-being

**DOI:** 10.1192/j.eurpsy.2024.1414

**Published:** 2024-08-27

**Authors:** E. Abdelmoula, B. Abdelmoula, N. Bouayed Abdelmoula

**Affiliations:** ^1^LR AMC, Ecole Doctorale Sciences et Ingénierie Architecturales (ED-SIA), Tunis; ^2^Genomics of Signalopathies at the service of Precision Medicine - LR23ES07, Medical University of Sfax, Sfax, Tunisia

## Abstract

**Introduction:**

While the acoustic environment in the cities correlates with various health-related problems, health benefits of natural sounds are proven. These positive effects of the sounds of nature should probably be taken seriously in urban design and urban renewal projects.

**Objectives:**

The aim of this study was to review the paradigm of natural soundscapes in the cities, psychological effects of natural soundscapes and the potential urban recommendations for such architecture design.

**Methods:**

We conducted a comprehensive review of the scientific literature using Web databases with the following keywords: natural soundscapes, natural sound, urban design, and mental health.

**Results:**

Our research found that improving the urban environment soundscape for the well-being of city dwellers has become one of the most pressing challenges of modern times. In a growing number of published studies, positive psychological effects of natural soundscapes are explored using various methods such as questionnaires, biofeedback sensors coupled with virtual reality experiences in laboratories, and quantification of the prevalence of restorative acoustic environments in parks. In a recent study (2023), Jian Kang from the United Kingdome, reported that “by taking psycho-acoustical, neural and physiological, and contextual factors into account, the European Research Council Soundscape Indices project will adequately reflect levels of human comfort, to integrate side-by-side with (and eventually replace) decibel-based metrics into existing (international) regulations”. The same paper highlighted how the transition from fighting noise pollution to creating soundscapes is key.

**Conclusions:**

Architects should develop mandatory guidelines regarding the spatial planning focusing on managing natural soundscapes in cities. Various sites such as green urban public spaces that offers exposure to natural sounds should be an integral part of the urban environment. These areas must be with a high abundance of natural sound (geophony and bio phony) and a low anthropogenic sound to enhance human physical and psychological health.

**Disclosure of Interest:**

None Declared

